# Risk Factors and Severity of Gastrointestinal Parasites in Selected Small Ruminants from Malaysia

**DOI:** 10.3390/vetsci7040208

**Published:** 2020-12-18

**Authors:** Bura Thlama Paul, Faez Firdaus Abdullah Jesse, Eric Lim Teik Chung, Azlan Che’Amat, Mohd Azmi Mohd Lila

**Affiliations:** 1 Department of Veterinary Clinical Studies, Faculty of Veterinary Medicine, Universiti Putra Malaysia, Serdang 43400, Selangor, Malaysia; bpaulgadzama@unimaid.edu.ng (B.T.P.); c_azlan@upm.edu.my (A.C.); 2 Veterinary Teaching Hospital, Faculty of Veterinary Medicine, University of Maiduguri, PMB 1069 Maiduguri, Borno, Nigeria; 3 Institute of Tropical Agriculture and Food Security, Universiti Putra Malaysia, Serdang 43400, Selangor, Malaysia; ericlim@upm.edu.my; 4 Department of Animal Science, Faculty of Agriculture, Universiti Putra Malaysia, Serdang 43400, Selangor, Malaysia; 5 Department of Veterinary Pathology and Microbiology, Faculty of Veterinary Medicine, Universiti Putra Malaysia, Serdang 43400, Selangor, Malaysia; azmi@upm.edu.my

**Keywords:** goats, coccidia, cestodes, strongyles, risk-factors, EPG, PCV, Malaysia

## Abstract

The productivity of smallholder sheep and goat flocks is constrained by high morbidity and mortality of young stock due to helminthosis and coccidiosis. We hypothesized that gastrointestinal parasites are prevalent and may cause severe infections amongst small ruminants in Malaysia. A cross-sectional survey was conducted between March and December 2019 to investigate the prevalence, risk factors, and levels of infection with gastrointestinal strongyle and coccidia in selected smallholder goat flocks in Negeri Sembilan, Malaysia. A total of 257 blood and fecal samples and management data were collected from four farms in Negeri Sembilan. Gastrointestinal parasites were detected by routine sodium chloride floatation, and the McMaster technique was used to quantify the fecal eggs/oocysts per gram outputs (EPG/OPG). The severity of infection was classified as mild (50–799), moderate (800–1200), or severe (>1200). The packed cell volume (PCV) was determined by microhematocrit centrifugation and classified as anemic or non-anemic. Coprological examination revealed an overall prevalence of 78.6% (CI = 72.74–83.44) and 100% flock level prevalence of strongyle and coccidia infection among goats from Negeri Sembilan with a higher infection in flock A-Lenggeng (95.6%) than B-Senawang (87.3%), D-Mendom (80.6%), or C-Seremban (60.0%). The co-infections of strongyle + *Eimeria* (50.6; CI = 44.50 to 56.64) were more common than single infections of either strongyle (16.7%; CI = 12.66 to 21.78) or *Eimeria* (4.3%; CI = 2.41 to 7.50). Quantitative analysis has revealed different (*p* < 0.05) patterns of EPG/OPG in various categories of goats. In total, there were 49.8% mild, 8.6% moderate, and 13.6% severe infections of strongyle and 40.1% mild, 6.6% moderate, and 19.8% severe infections of coccidia among goats. The mean PCV of goats with severe strongyle infection (24.60 ± 0.85) was significantly (*p* < 0.05) lower than the moderate (26.90 ± 1.15), or mild (28.23 ± 0.50) infections and the uninfected (30.4 ± 0.71). There were increased odds of infection with strongyle and coccidia among female (OR = 3.2) and adult (OR = 11.0) goats from smallholder flocks in Negeri Sembilan. In conclusion, gastrointestinal strongyles and coccidia occur at high frequency among smallholder goats, and there is a higher risk of infection amongst the adult and female stock.

## 1. Introduction

Small ruminant production is an emerging and essential component of the Malaysian agricultural economy that provides jobs and animal protein [1]. The rapid development of the livestock industry is hampered by disease outbreaks and the cost of prevention and control [2,3]. Parasitism is a global phenomenon that is limiting the productivity of small ruminants due to its negative impact on their health and welfare [4,5].

Goats are affected by a wide range of gastrointestinal parasites (GIPs) such as protozoa, trematodes, cestodes, and nematodes [6]. Coccidiosis in sheep and goats is caused by several *Eimeria* species, of which *E. arloingi* and *E. ninakohlyakimovae* are the most pathogenic [7]. Coccidiosis is also recognized as an economic disease in small ruminants due to frequent diarrhea, reduced weight gain, and reduced milk production [8]. Among the GIPs, gastrointestinal nematodes, mainly the strongyles are the most pathogenic and economically-important species affecting small ruminants worldwide [4,9]. *Haemonchus contortus* is a widespread and significant species of strongyle distributed among sheep and goats worldwide [10]. Gastrointestinal infections usually occur as a mild subclinical disease in healthy animals, but the severe acute disease may be seen in young, malnourished, concurrently infected, or immunocompromised animals [11]. The acute illness in sheep and goats is manifested by diarrhea, anemia, reduced weight gain, weakness, oedema in the lower parts, decreased productivity and occasional mortality [7].

Diseases caused by helminths, coccidia, and hemoparasites are associated with illness and economic losses in small ruminant production in the tropics [12,13,14,15]. The direct consequences of GIP infection on small ruminant production in the tropics include morbidity, mortality, and the cost of treatment and control measures [12,16,17]. Early research work has shown that GIP infections due to helminthosis and coccidiosis are widespread amongst small ruminants in Malaysia [14,18,19,20]. Moreover, *H. contortus* is responsible for high morbidity and mortality in smallholder flocks in Malaysia [21]. Despite the high prevalence of GIPs reported in other states and potential economic concerns in the Malaysian small ruminant industry, there is a dearth of published information on its epidemiology in Negeri Sembilan. It was hypothesized that gastrointestinal parasites are prevalent and cause severe infections among various groups of small ruminants in Malaysia. This study was therefore designed to investigate the prevalence, risk factors, and levels of infection with gastrointestinal strongyles and coccidia in selected smallholder goat flocks in Negeri Sembilan, Malaysia.

## 2. Materials and Methods

### 2.1. Ethical Approval and Consent

This study design was approved by the Institutional Animal Care and Use Committee (IACUC), Universiti Putra Malaysia (UPM/IACUC/AUP-R041/2019). The sampling and data collections from farms were approved by the Director-General Veterinary Services (DVS) Malaysia. We also obtained the list of all small ruminant farms from the DVS Negeri Sembilan and contacted individual farmers to obtain written consent for participation in the study.

### 2.2. Study Area

**Figure 1:**
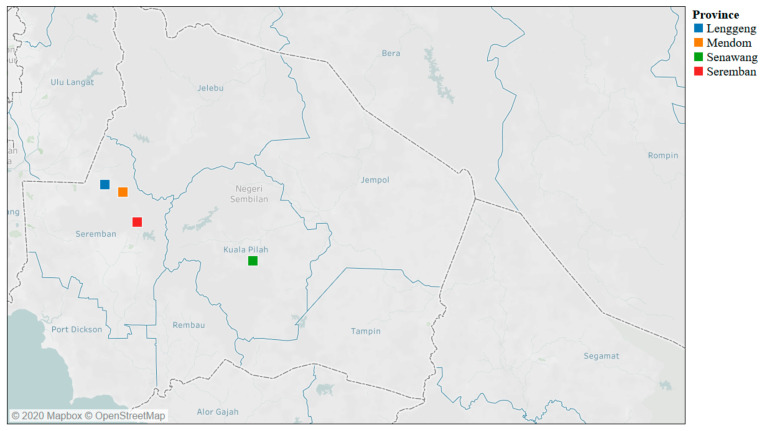
Map of Negeri Sembilan showing the study area.

Negeri Sembilan state is located on the southwest coast of Peninsular Malaysia between latitude (2°45′00.0” N) and longitude (102°15′00.0” E). The state has total 63,673 small ruminants distributed among individual smallholder and government farms within the seven districts of the state. We sampled one smallholder in four provinces from two districts within the state; Mendom (2°50′51.7” N, 101°57′43.0” E) in the Kuala Pilah district, and Lenggeng (2°51′47.0” N 101°55′27.6” E), Senawang (2°41′56.4” N, 101°58′32.1” E) and Seremban (2°47′04.8” N, 101°59′33.3” E) in the Seremban district as shown in Figure 1. The farms included in the study are smallholders who practiced semi-intensive management that allowed limited grazing during the day and fed supplementation in the evening.

### 2.3. Sample Size and Study Design

The sample size for this study was calculated according to [22] based on a large population, the assumptions were 88% expected prevalence (XP) from a previous study [23], 5% desired absolute precision (d), and Z = 1.96^2^ at 95% confidence interval. Thus, n = Z^2^. XP (1-XP)/d^2^ = 162, but we collected 257 samples to increase precision. A cross-sectional survey design was adopted in this study to simultaneously collect samples and essential demographic and management data during a single farm visit. The four goat farms which took part in the study were visited once between April and December 2019, to collect blood and fecal samples along with environment and management information. Before sampling, we conducted a physical examination on each animal to determine gender, age, breed, reproductive status, FAMACHA© eye score, and body condition score. Animals were aged by dentition and grouped as young (<1 year) and adults (one year and above), while gender and breed were determined using phenotypic characteristics. Body condition score (BCS) was assessed by palpation in the lumbar and sternum region and animals were classified as emaciated (1), thin (2), average (3), fat (4), or obese (5), respectively [24]. The ocular mucous membrane of individual animal was examined once during sampling and classified based on the Faffa Malan eye color chart (FAMACHA©) as 1 = red (non-anemic), 2 = red-pink (non-anemic); 3 = pink (mildly-anemic), 4 = pink-white (anemic), and 5 = white (severely anemic) [25].

### 2.4. Sample and Data Collection

Approximately 5 mL of the blood sample was collected from the jugular vein using heparinized vacutainer tubes and kept in a cold box at 4 ℃ during transportation. At the same time, we collected about 5 g of feces per rectum using clean gloves and kept this in plastic tubes containing 5% formalin as a preservative. The farmers also completed a structured questionnaire to provide farm-management data.

### 2.5. Laboratory Examinations

#### 2.5.1. Detection of GIPs and Evaluation of Fecal Egg/Oocyst Count

The qualitative fecal analysis was performed using saturated sodium chloride (400 g NaCl in 1000 mL of water; specific gravity 1200) floatation technique [4]. The crushed fecal pellets were suspended in saturated NaCl and sieved through a tea strainer. The filtrate was then poured into 10 mL floatation bottles, coverslips were applied, and it was left to stand for 10 min. The coverslips were mounted on glass slides and examined under 100 magnification for morphological identification of GIP eggs/oocysts, according to [7]. The positive samples were subjected to quantitative analysis using a simple McMaster technique with a sensitivity of 50 eggs/oocysts per gram feces to determine the strongyle/coccidia egg/oocyst per gram outputs [4]. In this test, 2 g of the fecal sample was homogenized in 28 mL of NaCl solution and sieved with a tea strainer to remove debris. The two chambers of a McMaster slide were filled with resultant fecal slurry using a Pasteur pipette and left undisturbed for 5 min. Microscopic examination was done using 100 magnification to count the total number of eggs/oocysts inside the grid areas in both chambers. Strongyle EPG and coccidia OPG were calculated separately as the total number of eggs/oocysts present in both chambers (C1 + C2) x 50, where 50 is a factor specific for the 2:28 feces/floatation solution ratio. The severity of strongyle/coccidia infection was reported based on egg/oocyst count according to [4] as mild (50–799EPG/OPG), moderate (800–1200EPG/OPG), or severe (>200EPG/OPG).

#### 2.5.2. Evaluation of Anemia

The packed cell volume (PCV) was determined by microhematocrit centrifugation (MHC) technique described by [26] and categorized animals based on per cent PCV as anemic (PCV < 22% in goat, PCV < 27% in sheep) or non-anemic (PCV > 22% in goat, PCV > 27% in sheep) [27].

### 2.6. Statistical Analysis

The prevalence of GIPs and corresponding 95% confidence intervals (CI) was calculated using EpiTools^®^ statistical calculators [28]. Data were summarized in Microsoft Excel^®^ spreadsheet program and analyzed with Statistical Package for Social Sciences Software (SPSS) Version 22.0. Univariable analysis by Chi-square test was performed to evaluate risk factors at a 5% level of significance (*p* < 0.05) using the status of parasitic infection as the dependent variable and the epidemiological factors as the independent variables. Significant variables (*p* < 0.05) in the univariable analysis were used to construct a forward stepwise (conditional) multivariable logistic regression model to calculate the adjusted odds ratios (AOR) at 5% level of significance. The mean and standard error of EPG, OPG, and PCV were calculated using one-way analysis of variance (ANOVA) and compared by Tukey’s HSD test at 5% level of significance.

## 3. Results

### 3.1. The Overall Prevalence and Spectrum of Parasites Detected among Small Ruminants from Negeri Sembilan, Malaysia

The qualitative fecal analysis revealed 81.32% (CI = 76.11 to 85.61) overall prevalence of parasitic infection among goats examined in Negeri Sembilan. There was a 100% herd-level prevalence of infection with the highest prevalence among goats from flock A-Lenggeng (95.6%), followed by B-Senawang (87.3%), D-Mendom (80.6%), and C-Seremban (60.0%) (Table 1). Co-infections of strongyle + *Eimeria* (50.6; CI = 44.50 to 56.64) were more common than single infections of either strongyle (16.7%; CI = 12.66 to 21.78) or *Eimeria* (4.3%; CI = 2.41 to 7.50). Mixed infections of strongyle + *Eimeria* + *Moniezia* (7.0%; CI = 4.47 to 10.79), Strongyle + *Moniezia* (0.8%; CI = 0.21 to 2.80), strongyle + *Trichuris* (0.4%; CI = 0.07 to 2.17), *Eimeria* + *Moniezia* (1.2%; CI = 0.40 to 3.38), and strongyle + *Eimeria* + *Trichuris* (0.4%; CI = 0.07 to 2.17) were also detected (Table 2). 

### 3.2. The Intensity of Parasitic Infection among Small Ruminants from Negeri Sembilan, Malaysia

The quantitative fecal analysis revealed that mean EPG was significantly higher (*p* < 0.05) among the breed of Saanen (1345.83 ± 322.71) than Boer goat (697.2 ± 73.9) and the mean OPG was significantly different (*p* < 0.05) between Saanen (7691.38 ± 2848.90) and Boer goat (1072.89 ± 122.63). Sex-wise, the mean EPG was significantly higher (*p* < 0.05) in the female (858.50 ± 1108.07) than male (1204.6 ± 244.9), but the mean OPG was significantly higher (*p* < 0.05) in male (6520.31 ± 2435.04) than female (1199.64 ± 263.12). In terms of age, the mean EPG was similar, but there was a significant (*p* < 0.05) higher mean OPG in young (13980.00 ± 5026.60) than adult (1062.18 ± 1517.32). According to their physiological status, the mean OPG output was significantly higher (*p* < 0.05) in immature (13980.00 ± 5026.60) than mating stock (1194.95 ± 161.64), lactating (862.00 ± 195.31), or pregnant (631.82 ± 204.85) goats but the mean EPG was not different (*p* > 0.05). Based on the body condition score, the mean EPG was significantly higher (*p* < 0.05) in thin (1488.89 ± 362.63) than average (676.43 ± 69.37), or fat (526.47 ± 112.22) goats. According to different FAMACHA^©^ Scores, there was a significantly-lower (*p* < 0.05) OPG count in anemic (954.81 ± 124.26) than in mildly anemic (4182.54 ± 1366.88), or severely-anemic (3150.00 ± 2426.16) goats. On the other hand, there was a significantly-higher (*p* < 0.05) EPG in goats with anemia based on PCV (1097.30 ± 193.18) than in non-anemic goats (702.38 ± 83.95). Among the different flocks, there was a significantly-lower (*p* < 0.05) mean EPG in C-Seremban (317.9 ± 52.38) than in Lenggeng (951.45 ± 162.26), or Mendom (1169.64 ± 256.64). There was a significant difference (*p* < 0.05) in the mean EPG between dairy (1345.322.71 ± 322.71) and meat (697.19 ± 73.99) goats (Table 3).

### 3.3. Effect of Different Levels of Strongyle Infection on the PCV of Small Ruminants from Negeri Sembilan, Malaysia

**Figure 2:**
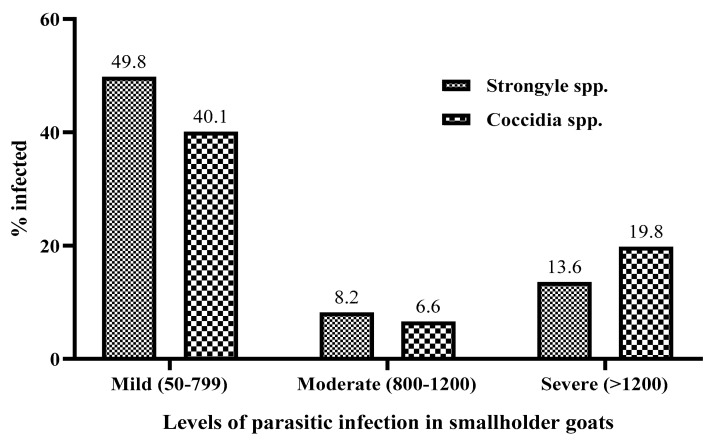
Prevalence of different levels of parasitic infection among goats from Negeri Sembilan, Malaysia.

**Figure 3:**
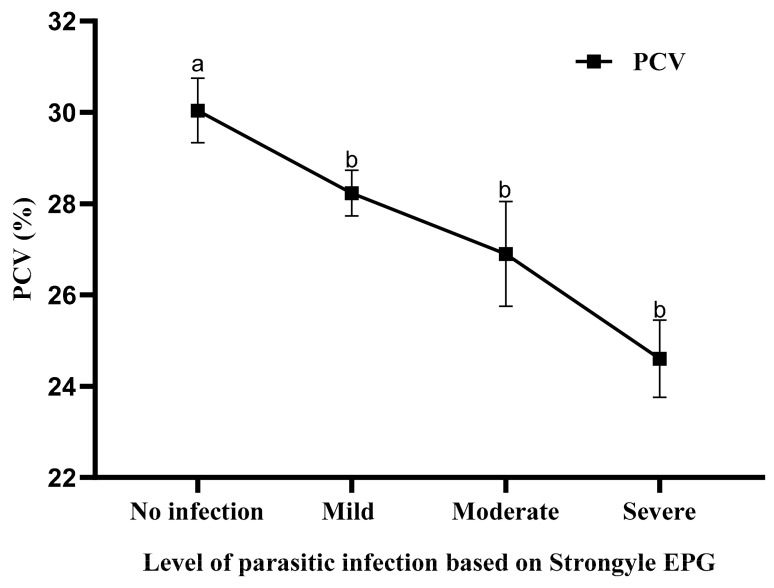
Effect of different levels of strongyle infection on the PCV of goats from Negeri Sembilan, Malaysia (PCV with different letters (a, b) are significantly different (*p* < 0.05)).

The quantitative analysis has also revealed that out of the total 209 (81.32%) positive animals, 128 (49.6%), 21 (8.2%), and 35 (13.6%) had a mild, moderate, or severe infection of strongyle, respectively. Out of a total 171 (66.5%) positive animals, 103 (40.1%), 17 (6.6%), and 51 (19.8%) had a mild, moderate, or severe coccidia infection, respectively (Figure 2). The mean PCV of goats with severe strongyle infection (24.60±0.85) was significantly lower (*p* < 0.05) than the moderate (26.90±1.15) and mild (28.23±0.50) infections and the uninfected (30.4±0.71) (Figure 3).

### 3.4. Risk Factors of GIP Infection Among Small Ruminants from Negeri Sembilan, Malaysia

The results of univariable analysis shows that breed (χ2 = 8.695, *p* = 0.003), gender (χ2 = 26.921, *p* = 0.000), age (χ2 = 55.221, *p* = 0.000), physiological status (χ2 = 48.547, *p* = 0.000), FAMACHA score (χ2 = 8.952, *p* = 0.030), flock (χ2 = 35.967, *p* = 0.001), and production purpose (χ2 = 8.695, *p* = 0.003) were associated with the risk of parasitic infection among goats from Negeri Sembilan, Malaysia (Table 4).

The results of multivariable logistic regression analysis further showed that female gender (OR = 3.1882; 95% CI = 1.41 to 7.19; *p* = 0.005) and adult age (OR = 11.007; 95% CI = 4.81 to 25.22; *p* = 0.000) were significantly associated with an increased odds of infection with common GIPs amongst goats from Negeri Sembilan, Malaysia. Thus, female goats were 3.2-times more likely to be infected with common GIPs than males and adults were 11-times more likely to be infected with common GIPs than young (Table 5).

## 4. Discussion

At the beginning of this study, it was hypothesized that gastrointestinal coccidia and strongyle parasites were prevalent and uniformly distributed among various categories of goats in the study area. This study investigated the prevalence, risk factors, and levels of infection with gastrointestinal coccidia and strongyle parasites in selected smallholder goat farms in the southwest coast of Peninsula Malaysia. Our results showed that 78.6% of sampled goats in all the farms were infected with various species of coccidia, cestode, and strongyles. The high prevalence of parasites recorded in this study is explained by the environment and management practices. High tropical temperatures, humidity, and rainfall are known to favor the epidemiology of parasites by ensuring optimal development of pre-parasitic stages of helminths and coccidia [7]. In the past, high prevalence of gastrointestinal parasites in the study area was associated with the humid tropical environment which ensures survival and availability of larval stages of pathogenic nematodes on pasture [2,29].

Moreover, most farmers in the study area allowed partial grazing on pastures around the edges of forests which favors reinfection due to pasture contamination. Furthermore, most of the farms under investigation did not practice routine deworming or prophylactic treatment against blood parasites and ectoparasites, which are known to increase the risk of infection with gastrointestinal parasites in small ruminants due to immunosuppressive effects of concurrent infections. For instance, concurrent hemotropic *Mycoplasma ovis* infection is known to exacerbate GIP infection in sheep and goats [3,30,31]. Hemoplasmas may act in synergy with highly-pathogenic nematodes such as *H. contortus* and contribute to the severity of parasitic gastroenteritis (PGE) in a concurrently-infected flock [32,33]. The overall prevalence of parasitic infection recorded in this study is almost similar to the results of previous studies in Malaysia [2,14,23]. However, our result is different from the 69.6% reported by [12] in small ruminants in Ethiopia, the 62.1% prevalence among goats in Bangladesh [34], and the 72% prevalence among sheep and goats in Nigeria [6]. The observed discrepancies could be explained by geographical differences in the prevalence of parasites due to the influence of various climatic factors [35].

Based on egg and oocyst morphology, the most prevalent species of parasites encountered in this study area were strongyle and *Eimeria*, with a few instances of *Moniezia* and *Trichuris* species. The current variety of parasites identified in goats is similar to that reported in previous studies in Malaysia [2,14,21,23]. Strongyle nematodes, especially *Haemonchus* and *Trichostrongylus,* are regarded as the most pathogenic species causing anemia and hypoproteinemia in sheep and goat flocks in the country [18,36,37]. *Oesophagostomum*, *Trichuris*, *Strongyloides* and *Eimeria* species were also recorded in small ruminants elsewhere [34,38,39]. The abundance of strongyle and *Eimeria* species in the study area has been linked to grazing in semi-intensive management, and a favorable humid climate, which ensures succession of parasites [2,29].

Strongyle fecal egg count (FEC) is a vital indicator of parasitic load and degree of pasture contamination [40]. The present difference in prevalence and intensity of infection amongst various breeds of goats concurs with previous reports [6,15,23]. This phenomenon is correlated with genetic and environmental factors which determine breed resistance or susceptibility to infectious diseases [22]. The observed difference in EPG outputs of goats kept on different farms reflects some differences in semi-intensive management practices of smallholders. The farms under study were isolated from each other and had different standards of hygiene, frequency of grazing, and frequency of deworming and prophylaxis, and hence different levels of pasture contamination and infection rates.

The presence of significantly-higher FEC in goats with a thin-body-condition score agrees with previous studies [6,41] and reflects the pathogenic effects of coccidia and strongyles. Infection of small ruminants with coccidia and strongyles leads to gastroenteritis, protein-losing enteropathy, poor weight gain, and loss of body condition [7,41,42]. The higher prevalence of parasites amongst dairy goats which had 100% infection rates is because milk-producing goats were the oldest category of animals on the farm. Older animals are usually more prone to GIP infection due to weak immunity [39]. Besides, most of the farms were located close to a water body which creates suitable parasite ecology and a high level of pasture contamination by grazing animals in the nearby forest and water pools.

This study also showed an association between the levels of strongyle infection and the PCV of goats such that significantly-lower PCV was recorded in animals with severe fecal egg counts. This finding agrees with previous reports [7,14,18] and is pathologically significant because strongyle nematodes such as *Haemonchus* and *Trichostrongylus* are known to cause anemia and hypoproteinemia in sheep and goats due to chronic intestinal hemorrhage and direct blood-feeding [18]. It is known that strongyle nematodes such as *Haemonchus* produce high fecal egg outputs, anemia, poor condition, and mortality, especially in young animals [7]. This finding was previously linked to the absence of active immunity to strongyle infection in young naïve kids and lambs [18]. Therefore, a heavy worm burden is likely to affect the productivity of smallholder goats in the study area due to anemia and protein loss.

The high prevalence of *Eimeria* oocysts among small ruminants in the current study is similar to the results of previous studies in Malaysia [14,43]. According to [44], oocysts of *Eimeria* species are commonly observed in the feces of small ruminants and are one of the economically-important diseases causing retarded growth and poor condition in young animals. The significant fecal oocyst count (FOC) observed among young goats in our study is similar to [45], who reported higher FOC in goat kids than adult females in Gran Canaria Island, Spain. This finding may be because young animals are not immune to infection and have an increased risk of exposure to oocysts shed by the dam [43]. Also, adult animals usually develop resistance and thrive well in the presence of coccidia without shedding high numbers of oocysts [44]. We also observed inadequate hygiene conditions around animal pens due to pillage of dung which may contaminate adjacent vegetation on which the animals may feed and be reinfected. Similarly, we associate the high intensity of coccidia infection among dairy goats to old age and feeding of fermented soya bean meal as a feed supplement and low hygiene standards on farms. Fermented soybean meal used in feeding dairy animals in the study area is an industrial by-product which is likely to be contaminated during packaging, transportation, and feeding of animals on the farm. Moreover, the environment of the farm, especially the feed storage and pen areas are unkempt, and feed may be easily contaminated with animal droppings, which facilitates the maintenance of coccidia infection in the herd.

The only tapeworm identified in our study was *Moniezia* species, an Anoplocephalid cestode of ruminants which is transmitted by various species of oribatid mites in the tropics [46]. The presence of *Moniezia* eggs may be explained by the preponderance of multiple species of oribatid mites in Peninsular Malaysia [47,48]. But, the low prevalence of *Moniezia* eggs may be due to low sensitivity of fecal floatation examination in detecting Cestode ova [49]. Furthermore, the low incidence of *Trichuris* eggs in our study is similar to the previous report in Kenya [50]. This finding may be due to difficulty in detecting light infection of *Trichuris* by the fecal floatation technique. The presence of higher proportions of mild strongyle and coccidia infections depicts a typical field infection where many animals were shedding a few oocysts/eggs because of adaptive immunity. This outcome is essential epidemiologically because it indicates that there are many carrier animals which always shed gastrointestinal parasites to contaminate pasture in the study area.

According to [51], the risk of GIP depends on parasite, host, and environmental factors. The associations between the prevalence of coccidia and strongyle infection and the gender and age of goats observed in this study agrees with the results of previous studies [10,39]. Usually, when adult goats are kept for long periods on the farm, they may acquire cumulative exposure and develop an adaptive response to thrive in the presence of a heavy worm burden. According to [39], the risk of infection was highest at the extreme ages (1.6 years), moderate at 2, 3, 5 years, and lowest at the age of 4 years. We have seen from our results that females were 3.2-times more at risk of coccidia and strongyle infection than males which is contrary to [39] who reported 2.8-times higher risk of GIP infection among male goats under extensive management systems in Zimbabwe. This finding is because, in the present study, females dominated the population of animals in smallholder farms. Moreover, the stress of production, such as pregnancy and lactation, may modify immunity and increase the susceptibility of females to disease.

## 5. Conclusions

In conclusion, mild infections of coccidia and strongyle are widespread amongst smallholder goat flocks examined in Negeri Sembilan. There was an increased risk of coccidia and strongyle infection in female and adult goats kept under traditional smallholder management system in Malaysia. The practice of routine deworming, provision of adequate nutrition, and strict environmental hygiene will help in minimizing production losses due to coccidia and strongyle infections in the smallholder farms.

## Figures and Tables

**Table 1:** The overall prevalence of parasitic infection among smallholder goats from Negeri Sembilan, Malaysia.

Factor	Examined	Prevalence (%)	95% CI *
**Breed**			
Saanen goat	33	33 (100)	89.57–100.00
Boer goat	224	176 (78.6)	72.74–83.44
**Gender**			
Male	68	41(60.3)	48.41–71.07
Female	189	168 (88.9)	83.61–92.62
**Age**			
Young	54	25 (46.3)	33.69–59.40
Adult	203	184 (90.6)	85.84–93.93
**Physiological Status**			
Immature	52	25 (48.1)	35.11–61.32
Mating stock	148	131 (88.5)	82.37–92.70
Lactating	30	29 (96.7)	83.33–99.41
Pregnant	27	24 (88.9)	71.94–96.15
**Body Condition Score**			
Fat	22	18 (81.8)	61.49–92.69
Average	201	162 (80.6)	74.58–85.47
Thin	34	29 (85.3)	69.87–93.55
**FAMACHA© Score**			
Severely anemic	07	06 (85.7)	48.68–97.43
Mildly anemic	88	73 (83.0)	73.76–89.39
Anemic	160	130 (81.3)	74.50–86.54
Non-anemic	02	00 (0.0)	0.00–65.76
**^1^ PCV Categories**			
Non-Anemic	211	171 (81.0)	75.21–85.76
Anemic	46	38 (82.6)	69.28–90.92
**Flocks**			
A-Lenggeng	91	87 (95.6)	89.23–98.28
B-Senawang	55	48 (87.3)	75.98–93.69
C-Seremban	75	45 (60.0)	48.69–70.34
D-Mendom	36	29 (80.6)	64.98–90.25
**Production Purpose**			
Dairy	33	33 (100)	89.57–100
Meat	224	176 (78.6)	72.74–83.44
**Overall**	257	209 (81.32)	76.11–85.61

* CI: 95% confidence interval, ^1^ packed cell volume (PCV) category (Jackson & Cockcroft, 2002).

**Table 2:** The spectrum of parasites among smallholder goats from Negeri Sembilan, Malaysia.

Categories of Infection	Positive	Prevalence (%)	95% CI *
Strongyle eggs	43	16.7	12.66–21.78
*Eimeria* oocysts	11	4.3	2.41–7.50
Strongyle + *Eimeria*	130	50.6	44.50–56.64
Strongyle + *Eimeria* + *Moniezia*	18	7.0	4.47–10.79
Strongyle + *Moniezia*	02	0.8	0.21–2.80
Strongyle + *Trichuris*	01	0.4	0.07–2.17
*Eimeria* + *Moniezia*	03	1.2	0.40–3.38
Strongyle + *Eimeria* + *Trichuris*	01	0.4	0.07–2.17

* CI: 95% confidence interval.

**Table 3:** The intensity of parasitic infection among small ruminants from Negeri Sembilan, Malaysia.

Factor	EPG (Mean ± SE)	OPG (Mean ± SE)
**Breed**		
Saanen goat	1345.83 ± 322.71 ^a^	7691.38 ± 2848.90 ^1^
Boer goat	697.19 ± 73.99 ^b^	1072.89 ± 122.63 ^2^
**Gender**		
Male	403.23 ± 120.86 ^a^	6520.31 ± 2435.04 ^1^
Female	858.50 ± 1108.07 ^b^	1199.64 ± 263.12 ^2^
**Age**		
Young	726.47 ± 234.84	13980.00 ± 5026.60 ^1^
Adult	787.43 ± 82.90	1062.18 ± 1517.32 ^2^
**Physiological Status**		
Immature	726.47 ± 234.84	13980.00 ± 5026.60 ^1^
Mating stock	707.46 ± 101.79	1194.95 ± 161.64 ^2^
Lactating	1177.59 ± 227.75	862.00 ± 195.31 ^2^
Pregnant	695.83 ± 131.60	631.82 ± 204.85 ^2^
**Body Condition Score**		
Fat	526.47 ± 112.22 ^a^	733.33 ± 243.91
Average	676.43 ± 69.37 ^a^	2350.00 ± 653.83
Thin	1488.89 ± 362.63 ^b^	2258.33 ± 957.76
**FAMACHA^©^ Score**		
Severely anemic	1416.67 ± 483.51	3150.00 ± 2426.16 ^1^
Mildly Anemic	793.44 ± 143.14	4182.54 ± 1366.88 ^1^
Anemic	743.16 ± 94.40	954.81 ± 124.26 ^2^
**^1^ PCV Categories**		
Non-Anemic (PCV > 22%)	702.38 ± 83.95 ^a^	2302.86 ± 594.33
Anemic (PCV ≤ 22%)	1097.30 ± 193.18 ^b^	1709.68 ± 1069.38
**Flocks**		
A-Lenggeng	951.45 ± 162.26 ^a^	3639.10 ± 1111.78
B-Senawang	713.33 ± 78.04	863.83 ± 130.75
C-Seremban	317.86 ± 52.38 ^b^	693.48 ± 261.01
D-Mendom	1169.64 ± 256.64 ^a^	1521.74 ± 531.43
**Production purpose**		
Dairy	1345.322.71 ± 322.71 ^a^	7691.38 ± 2848.90 ^1^
Meat	697.19 ± 73.99 ^b^	1072.89 ± 122.63 ^2^

Superscripts (^a, b^ & ^1, 2^): denotes significant difference (*p* < 0.05): ^1^ PCV category (Jackson & Cockcroft, 2002).

**Table 4:** Univariable analysis for risk factors of parasitic infection amongst small ruminants from Negeri Sembilan, Malaysia.

Factor	Number	Positive	χ2	p
**Breed**				
Saanen goat	33	33 (100)	8.695	0.003 *
Boer goat	224	176 (78.6)		
**Gender**				
Male	68	41(60.3)	26.921	0.000 *
Female	189	168 (88.9)		
**Age**				
Young	54	25 (46.3)	55.221	0.000 *
Adult	203	184 (90.6)		
**Body Condition Score**				
Fat	22	18 (81.8)	0.426	0.808
Average	201	162 (80.6)		
Thin	34	29 (85.3)		
**Physiological Status**				
Immature	52	25 (48.1)	48.547	0.000 *
Mating stock	148	131 (88.5)		
Lactating	30	29 (96.7)		
Pregnant	27	24 (88.9)		
**FAMACHA^©^ Score**				
Severely anemic	07	06 (85.7)	8.952	0.030 *
Mildly anemic	88	73 (83.0)		
Anemic	160	130 (81.3)		
Non-anaemic	02	00 (0.0)		
**^1^ PCV Categories**				
Non-anaemic	211	171 (81.0)	0.061	0.805
Anaemic	46	38 (82.6)		
**Flocks**				
A-Lenggeng	91	87 (95.6)	35.967	0.000 *
B-Senawang	55	48 (87.3)		
C-Seremban	75	45 (60.0)		
D-Mendom	36	29 (80.6)		
**Production Purpose**				
Dairy	33	33 (100)	8.695	0.003 *
Meat	224	176 (78.6)		

χ2: Chi-square, *: denotes significance (*p* < 0.05)**:** ^1^ PCV category (Jackson & Cockcroft, 2002).

**Table 5:** Multiple logistic regression of risk factors associated with gastrointestinal parasite (GIP) infection amongst small ruminants from Negeri Sembilan, Malaysia.

Risk Factor	β	SE	df	p	AOR	95% CI
**Gender** (Female)	1.16	0.42	1	0.005 *	3.1882	1.41–7.19
**Age** (Adult)	2.39	0.42	1	0.000 *	11.007	4.81–25.22

β: regression coefficient, SE: standard error, AOR: adjusted odds ratio, CI: 95% confidence interval, *: denotes significance (*p* < 0.05).
